# Shedding of APP limits its synaptogenic activity and cell adhesion properties

**DOI:** 10.3389/fncel.2014.00410

**Published:** 2014-12-03

**Authors:** Ronny Stahl, Sandra Schilling, Peter Soba, Carsten Rupp, Tobias Hartmann, Katja Wagner, Gunter Merdes, Simone Eggert, Stefan Kins

**Affiliations:** ^1^Center of Molecular Biology ZMBH, University of HeidelbergHeidelberg, Germany; ^2^Department of Physiological Genomics, Institute of Physiology, Ludwig-Maximilians University MunichMunich, Germany; ^3^Department of Human Biology and Human Genetics, Technical University of KaiserslauternKaiserslautern, Germany; ^4^Center for Molecular Neurobiology (ZMNH), University of HamburgHamburg, Germany; ^5^Deutsches Institut für DemenzPrävention, Experimental Neurology, Saarland UniversityHomburg/Saar, Germany; ^6^Department of Biosystems Science and Engineering, ETH ZürichBasel, Switzerland

**Keywords:** Alzheimer’s disease, amyloid precursor protein, APP processing, cell adhesion, SAM, synaptogenic activity

## Abstract

The amyloid precursor protein (APP) plays a central role in Alzheimer’s disease (AD) and has essential synapse promoting functions. Synaptogenic activity as well as cell adhesion properties of APP presumably depend on trans-cellular dimerization via its extracellular domain. Since neuronal APP is extensively processed by secretases, it raises the question if APP shedding affects its cell adhesion and synaptogenic properties. We show that inhibition of APP shedding using cleavage deficient forms of APP or a dominant negative α-secretase strongly enhanced its cell adhesion and synaptogenic activity suggesting that synapse promoting function of APP is tightly regulated by α-secretase mediated processing, similar to other trans-cellular synaptic adhesion molecules.

## Introduction

A new era of Alzheimer’s disease (AD) research began with identification of the Amyloid-β (Aβ) peptide as a major amyloid plaque component (Masters et al., 1985). Amyloid-β is derived from the amyloid precursor protein (APP; Kang et al., 1987) by sequential cleavages of β- and γ-secretase. In the amyloidogenic pathway, β-secretase (BACE1) cleavage releases the large ectodomain of APP (sAPPβ) while generating the membrane-anchored C-terminal APP fragment (β-CTF; Vassar et al., 1999). Cleavage of β-CTF by γ-secretase leads to the secretion of Aβ peptides of various lengths and the release of the APP intracellular domain (AICD) into the cytosol (Weidemann et al., 2002; Kakuda et al., 2006). Alternatively, APP can first be cleaved in the non-amyloidogenic pathway by α-secretase within the Aβ domain (Esch et al., 1990). In neurons, this cleavage is mainly mediated by the protease ADAM10 (Kuhn et al., 2010; Prox et al., 2013) and releases the APP ectodomain (sAPPα) while generating the membrane-bound C-terminal fragment (α-CTF). The latter can be further processed again by the γ-secretase complex, resulting in the secretion of a 3-kDa fragment (p3) and the release of AICD (Weidemann et al., 2002).

Amyloid precursor protein is part of a larger gene family, which includes two mammalian homologs, the amyloid precursor like protein 1 and 2 (APLP1 and APLP2; Walsh et al., 2007; Jacobsen and Iverfeldt, 2009). It has been shown that all APP family members can dimerize in a homo- and heterotypic manner in cis- and in trans-orientation (Soba et al., 2005; Kaden et al., 2009). All APP family members across different species including *D. melanogaster* (APPL) share similar domain architectures (Luo et al., 1990). Accordingly, the large extracellular domain contains the highly conserved E1 and E2 domains, which are connected by an acidic domain (Reinhard et al., 2005; Soldano and Hassan, 2014). The E1 domain was identified as the major interaction interface for homo- and hetero-dimerization of APP, APLP1 and APLP2 (Soba et al., 2005; Kaden et al., 2009; Dahms et al., 2010) suggesting a function of APP in cell adhesion (Herms et al., 2004; Young-Pearse et al., 2007).

Aged mice of APP single knockouts show impairment in spatial learning (Müller et al., 1994; Phinney et al., 1999; Ring et al., 2007) and long-term potentiation (Seabrook et al., 1999; Ring et al., 2007; Tyan et al., 2012). Furthermore, a reduced number of dendritic spines (Lee et al., 2010; Tyan et al., 2012; Weyer et al., 2014) and a reduced overall dendritic length in the CA1 region has been reported (Seabrook et al., 1999). APP/APLP2 double knockout (dko) mice die shortly after birth and display profound neuronal defects in the central and peripheral nervous system. Analysis of the neuromuscular junction (NMJ) revealed incomplete apposition of the pre- and postsynaptic structures (Wang et al., 2005), a reduced number of docked presynaptic vesicles and an impaired synaptic transmission (Wang et al., 2005). Mice that express only sAPPα in an APP/APLP2 dko background show less pronounced, but also severe defects in the peripheral as well as in the central nervous system, including motor and learning deficits (Weyer et al., 2011). This argues that sAPPα, although representing the major secreted species of APP, only partially rescues APP function. Notably, APP family members are expressed pre- and postsynaptically (Kim et al., 1995; Lyckman et al., 1998; Back et al., 2007; Hoe et al., 2009; Wang et al., 2009; Wilhelm et al., 2014), a prerequisite for synaptic adhesion molecules (Siddiqui and Craig, 2011; Baumkötter et al., 2012). A recent publication showed APP to be predominantly located at the surface of synaptosomes (Wilhelm et al., 2014). Further, tissue specific deletion of APP in either presynaptic motor neurons or postsynaptic muscle cells in APLP2−/− mice demonstrated similar NMJ defects as observed in APP/APLP2 dko mice (Wang et al., 2009). In conclusion neither sAPP nor expression of APP only at the pre- or postsynaptic site is sufficient for proper formation of the NMJ.

In line with these analyses, co-culture assays of a non-neuronal cell line seeded on primary neurons (Biederer and Scheiffele, 2007) revealed that expression of APP in non-neuronal cells promotes presynaptic differentiation of contacting axons (Wang et al., 2009; Baumkötter et al., 2014), similar to Neuroligin-1 (NLG-1; Scheiffele et al., 2000; Wang et al., 2009). Synapse promoting activity of APP in the hemisynaptic assay depends on expression of APP containing the E1 domain on both sides, similarly to what was shown for cell adhesion properties of APP (Soba et al., 2005; Wang et al., 2009; Dahms et al., 2010).

Recent publications suggest that the synaptogenic activity of synaptic adhesion molecules (SAM) is regulated by ectodomain shedding (Suzuki et al., 2012; Pettem et al., 2013). Since APP is heavily processed by secretases, we investigated the influence of proteolytic processing on trans-interaction properties of APP and its effect on APP synaptogenic function.

## Results

### Generation of secretion deficient APP mutants

**Figure 1:**
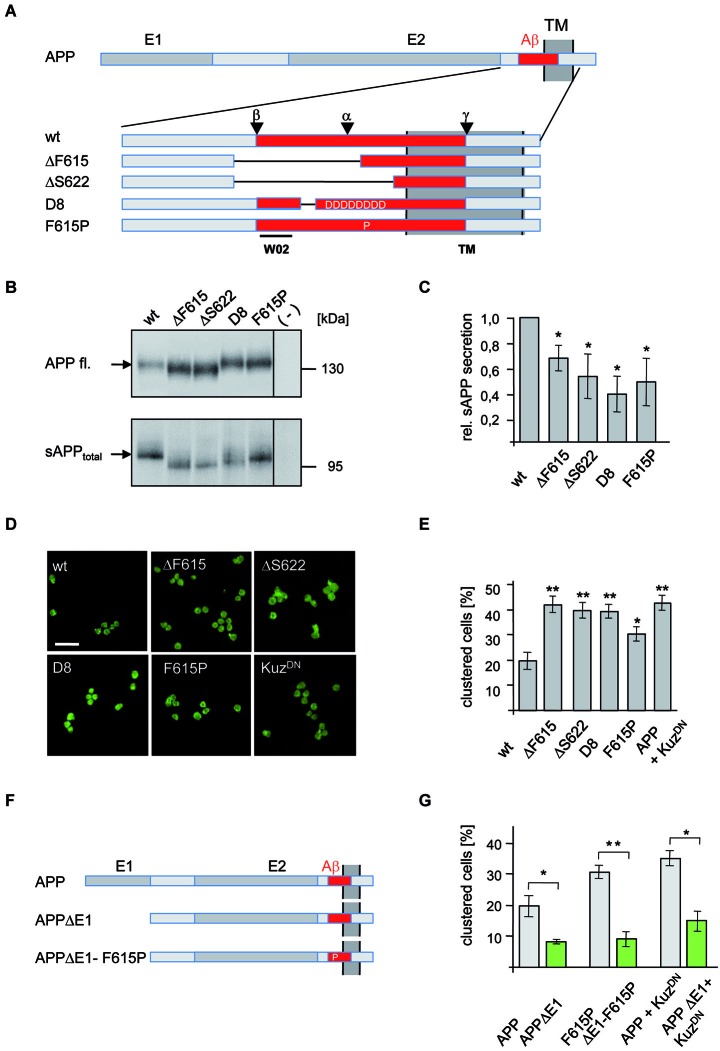
**Analysis of APP secretion deficient mutants in *Drosophila* S2 cells. (A)** Schematic representation of N-terminally myc tagged APP secretion deficient constructs. The E1 and E2 domain as well as the transmembrane (TM) domain are highlighted in dark gray. The Aβ region of APP (red) is shown enlarged and the position of deletions and amino acid substitutions is given. Additionally, positions of α-, β-, and γ-cleavage sites are indicated as well as the epitope of antibody W02 (aa 2–8). Deletions of the APP mutants are marked with lines. **(B)** Western Blot analysis of sAPP_total_ secretion from transfected S2 cells expressing APPwt and mutant variants. Direct load of cellular extracts and medium-IP (22734) samples were subjected to SDS-PAGE and immunoblotted with antibody 22C11. **(C)** Densitometric quantification of sAPP_total_/APP fl. ratio (*t*-test, *n* ≥ 3, ±SEM). **(D)** S2 cells, transiently transfected with APPwt or secretion deficient APP-mutants were aggregated and immunostained with an anti c-myc antibody. Scale bar: 20 μm. **(E)** Quantification of clustered wild type APP and mutant APP expressing S2 cells (*t*-test, *n* ≥ 5; ± SEM); (* *p* < 0.05; ** *p* < 0.001; *** *p* < 0.0001). **(F)** Scheme of APP mutants lacking the E1 domain. **(G)** S2 cells were transiently transfected with human APPwt or secretion deficient APP-F615P and the corresponding E1-deletion constructs (APPΔE1, APP-F615PΔE1) or co-transfected with APPwt or APPΔE1 and a dominant negative form of Kuzbanian (Kuz^DN^). Cells were aggregated and immunostained with anti c-myc antibody showing that secretion deficient APP cell-clustering depends on the E1-domain. Quantification of clustered cells given as percentage of the amount of transfected cells (*t*-test, *n* ≥ 5; ± SEM; * *p* < 0.05; ** *p* < 0.001; *** *p* < 0.0001).

We have previously shown using a Schneider (S2) cell based aggregation assay (Tsiotra et al., 1996; Klueg and Muskavitch, 1999; Islam et al., 2004) that APP possesses adhesion properties and can induce cellular aggregation (Soba et al., 2005). To investigate the consequences of α-secretase processing on APP-mediated cell adhesion, we designed different putative secretion deficient APP mutants: N-terminally myc-tagged APP carrying either an amino acid substitution (F615P) previously shown to lower α-secretase cleavage (Sisodia, 1992), small deletions removing the α-secretase and β-secretase cleavage site (APPΔF616, APPΔS622), and deletion of Aβ_10–24_ including amino acid substitutions with aspartates to increase electrostatic repulsion of α-secretase (APP-D8; Figure 1A).

To test shedding deficiency of these different APP mutants, S2 cells were transfected and sAPP_total_ was analyzed (Figure 1B). All investigated APP mutants showed a significant reduction in sAPP_total_ generation while cellular APP amounts were only moderately increased, likely due to reduced processing (Figure 1C). The strongest effect on sAPP_total_ secretion with an approximately 60% reduction was observed for APP-D8. These data suggest that deletion of the APP cleavage sites or interference with α-secretase substrate binding by electrostatic repulsion efficiently reduces APP processing.

### Secretion deficient APP accelerates cell clustering

To investigate if reduced APP processing affects cell adhesion, we next analyzed the secretion deficient mutants of APP in a previously used S2 cell aggregation assay (Soba et al., 2005; Figures 1D,E). Strikingly, all secretion-impaired forms of APP induced significantly increased cell clustering (Figures 1D,E). We observed a more than two-fold increase in clustered cells expressing the secretion-deficient constructs APP-D8, APPΔF616 and APPΔS622 compared to APPwt expressing cells. Consistent with its weaker inhibition of APP cleavage, the smallest increase in clustering-ability was observed in APP-F615P expressing cells. To further validate these results, we inhibited α-secretase cleavage by co-expression of a dominant-negative form of Kuz^DN^ (Pan and Rubin, 1997), the *Drosophila* ADAM10 homolog. Co-expression of Kuz^DN^ together with APP in S2 cells caused strongly increased cell clustering (Figures 1D,E), comparable to that observed with cells expressing secretion-deficient APP mutants. Together these data confirm our hypothesis that inhibition of α-secretase cleavage promotes APP-mediated trans-cellular adhesion.

### APP mediated cell adhesion properties are E1 domain dependent

The APP E1 domain is believed to be the major interface mediating the trans-interaction of APP as APP lacking the E1 domain (APPΔE1) displayed reduced cell adhesion properties (Soba et al., 2005) and lower synaptogenic activity (Wang et al., 2009).

In order to analyze whether increased APP mediated cell-cell interaction caused by inhibition of α-cleavage also depends on the E1 domain, we generated an APP mutant construct carrying both, a secretion deficient mutation and a deletion of the E1 domain (APPΔE1-F615P; Figure 1F). S2 cells were transiently transfected with APP, APPΔE1, APP-F615P or APPΔE1-F615P and allowed to aggregate after induction of APP expression. Quantification of clustered APP expressing cells revealed that APPΔE1 or APPΔE1-F615P induced significantly less cell clustering in comparison to wildtype APP (Figure 1G). Similarly, aggregated S2 cells co-expressing APPΔE1 together with Kuz^DN^ showed significantly decreased cell clustering in comparison to cells co-expressing APP and Kuz^DN^ (Figures 1F,G). These data validate our previous results showing that the E1 domain is required for APP mediated cell clustering. Moreover, it strongly suggests that increased cell clustering by inhibition of α-secretase cleavage depends on trans-dimerization properties of APP and not other potential cell adhesion molecules expressed in S2 cells.

### Processing of secretion deficient APP mutants in mammalian cells

**Figure 2:**
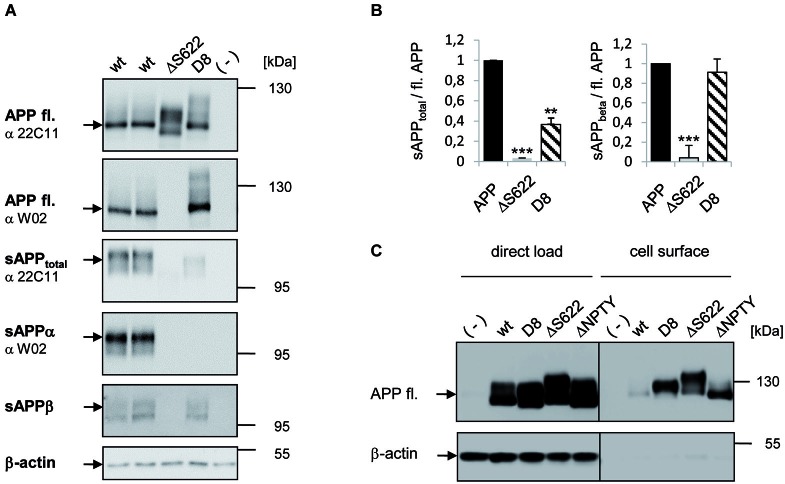
**Analysis of secretion deficient mutants in HEK293 cells. (A)** Western Blot analysis of APP secretion deficient mutants. HEK293 cells were transiently transfected with APPwt and mutants APPΔ622 and APP-D8. Cell lysates were analyzed with antibody 22C11 or W02 to detect full length APP via Western Blot. Medium samples of APP were analyzed with antibody 22C11 to detect sAPP_total_, antibody W02 to detect sAPPα and an sAPPβ specific antibody. Note, the W02 epitope is deleted in APPΔ622. β-actin antibody served as a loading control. **(B)** Quantification of data shown in panel **(A)**. One-way ANOVA followed by Tukey’s *post hoc* analyses (* *p* < 0.05; ** *p* < 0.001; *** *p* < 0.0001, *n* = 5 ± SEM). **(C)** Cell surface biotinylation of APP secretion deficient mutants. HEK293 cells were transfected with the indicated constructs. Direct load of cell lysates is documented in the left panel together with the β-actin loading control. In the right panel, APP cell surface levels after streptavidin immunoprecipitation and Western Blot detection with antibody c-myc is shown and β-actin as a negative control for intracellular proteins at the cell surface.

To investigate if inhibition of APP processing also promotes APP trans-interaction properties in mammalian cells, we first analyzed proteolytic processing of the APP mutants APPΔS622 and APP-D8 that displayed the strongest effect in *Drosophila* S2 cells (Figure 2). Expression of these APP forms in HEK293 cells followed by Western blotting detected appropriate expression (Figure 2A). Both APP mutations resulted in a highly significant reduction in sAPP_total_ secretion (*p* < 0.0001; Figures 2A,B). For APPΔS622, sAPP_total_ levels were even stronger decreased than for APP-D8. For APPΔS622 it is not possible to differentiate between sAPPα and sAPPβ secretion, as the corresponding region between the α- and β-cleavage sites was deleted. The low residual amount of sAPP_total_ in case of APP-D8 was mainly caused by an impairment of α-secretase cleavage, as shown by Western blot analysis with an antibody (W02) directed against the C-terminus of sAPPα (Ida et al., 1996; Figure 2A). W02 antibody binding to epitope 2–8 of the Aβ sequence (Miles et al., 2008; Figure 1A) was not affected by the APP-D8 mutation as shown for full length APP-D8 detection. Conversely, we observed no significant decrease in β-site cleavage for APP-D8 (Figures 2A,B). This indicates that exchanging amino acids surrounding the α-cleavage site to aspartate residues nearly completely abolishes α-secretase cleavage of APP without affecting β-secretase ectodomain shedding.

We hypothesized that the observed reduction in APP shedding should result in an increase of full length APP at the cell surface. Indeed, we detected strongly increased plasma membrane levels of shedding deficient APP-D8 and APPΔS622 by cell-surface biotinylation experiments (Figure 2C). Cell surface levels were increased to a similar degree when using an internalization deficient APP lacking the NPTY motif (Figure 2C), suggesting that both, cleavage and internalization of APP contribute to its turnover and plasma membrane levels.

Together, these data show that APP cleavage site mutagenesis efficiently suppresses secretion in mammalian cells as well and results in accumulation of APP at the cell surface.

### APP synaptogenic function depends on APP processing

**Figure 3:**
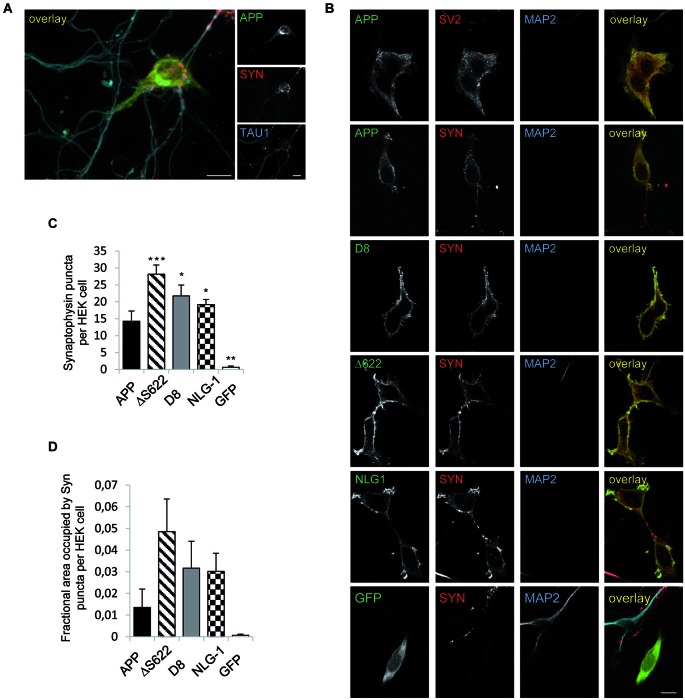
**APP processing deficient mutants show stronger synaptogenic activity than APPwt**. The synaptogenic activity of APP was analyzed in a co-culture assay. HEK293 cells were transiently transfected with APPwt and seeded 24 h later on primary cortical neurons (DIV7). The cells were fixed after 24 h (neurons DIV 8) and analyzed via immunocytochemistry. **(A)** Cells were stained with TAU1 as axonal marker, Synaptophysin as presynaptic marker and antibody c-myc for APP. **(B)** HEK293 cells were transfected with NLG-1 as a positive control, GFP as a negative control, APPwt and APP processing deficient constructs. Cells were stained with antibody MAP2 as a dendritic marker and Synaptophysin or SV2 as a marker for presynaptic vesicles and antibody c-myc to visualize heterologously expressed APP. **(C)** Quantification of Synaptophysin positive puncta per HEK293 cell. **(D)** Quantification of Synatophysin covered area per HEK293 cell. Bars represent mean values ± SEM of at least three independent experiments (*n* ≥ 3; one-way ANOVA; * *p* < 0.05, ** *p* < 0.01, and *** *p* < 0.005 compared to APP fl.).

In previous studies using a mixed-culture system (Scheiffele et al., 2000; Graf et al., 2004; Biederer and Scheiffele, 2007), it has been shown that expression of APP in HEK293 cells causes recruitment of contacting axons from co-cultured primary neurons (Wang et al., 2009). We reproduced this analysis and validated the reported synaptogenic activity of APP to induce presynaptic differentiation of contacting axons as indicated by clustering of synaptophysin and SV2 at TAU-positive axon terminals (Figures 3A,B). To investigate the synaptogenic properties of processing-deficient mutant forms of APP, we transiently transfected NLG-1, wild-type APP (APP fL), APP-D8, APPΔS622, as well as GFP in HEK293 cells. The transfected cells were then co-cultured with primary cortical neurons and assayed for their capacity to induce presynaptic differentiation. Neuroligin-1 expressing HEK293 cells served as a positive control (Scheiffele et al., 2000), while GFP expressing HEK293 cells were used as a negative control (Figure 3B). Similarly to NLG-1, expression of APP but not GFP potently promoted synaptic puncta formation as measured by the number of Synaptophysin positive puncta and area covered per transfected HEK293 cell (Figures 3C,D). Interestingly, both processing deficient mutant APP forms promoted presynaptic differentiation of contacting axons to a much higher extent than APP wild-type, which in case of APP-D8 was even stronger than the synaptogenic activity of NLG-1. Taken together, these data corroborate our hypothesis that APP synaptogenic function is limited by the extent of APP shedding activity.

## Discussion

Trans-cellular dimerization of APP is assumed to mediate cell adhesion and synaptogenic activity, similar to NLG-1 and other synaptic cell adhesion molecules (Siddiqui and Craig, 2011; Baumkötter et al., 2012; Müller and Zheng, 2012). Here, we show that inhibition of processing promotes APP-mediated cell adhesion and synaptogenic properties, indicating that APP shedding negatively regulates trans-dimerization dependent physiological functions of APP.

Once APP reaches the plasma membrane, it normally undergoes rapid proteolytic conversion by sheddases (Lammich et al., 1999; Kuhn et al., 2010; Prox et al., 2013). Therefore, we expected that inhibition of APP processing would enhance APP trans-interaction properties. To test this hypothesis we were using Schneider cells because of their semi-adherent characteristics and due to the fact that they don’t express APPL endogenously. Our analysis of APP-mediated cell adhesion, using the *Drosophila* S2-cell aggregation assay (Tsiotra et al., 1996; Islam et al., 2004; Soba et al., 2005) revealed that the extent of cell clustering increases with a decreased rate of mutant APP cleavage.

So far, the strongest inhibition of APP α-secretase cleavage has been achieved in mammalian cells by introducing single amino acid exchanges V614G and F615P (Sisodia, 1992). We tested the F615P mutant in *Drosophila* and human cells and could validate the reduction in sAPP secretion (Figure 2). Interestingly, the highest extent of sAPP suppression was observed for the APP-D8 and APPΔS622 mutants. In case of APP-D8, the mutation mostly affected α- but not β-secretase cleavage. In this case α-secretase activity, which has been shown to exhibit low sequence specificity (Maruyama et al., 1991; Sahasrabudhe et al., 1992; Sisodia, 1992; Zhong et al., 1994), might be inhibited by electrostatic repulsion due to the introduced aspartate stretch. This strategy has been successfully used before to prevent metalloprotease cleavage of the Notch ligand Delta 1 (Six et al., 2003). Our data suggest a tight correlation between APP shedding and APP mediated cell-cell interaction. These results were validated by inhibition of α-secretase activity using a dominant-negative variant of Kuz^DN^ (Pan and Rubin, 1997). Expression of Kuz^DN^ suppresses endogenous α-secretase activity and thus concomitantly increased cell clustering of APP-transfected S2 cells. Dominant-negative Kuzbanian may also affect other ADAM10 substrates like E-Cadherin or CX3CL1 (Hundhausen et al., 2003; Maretzky et al., 2005), which possibly modulate S2 cell adhesion independent of APP. To exclude that Kuz^DN^ expression elevated clustering of S2 cells in an APP independent manner, we used an APP construct lacking the E1 domain that is essential to mediate APP trans-cellular interactions. Amyloid precursor protein secretion deficient mutants containing the E1 deletion (APPΔE1) as well as co-expression of APPΔE1 with Kuz^DN^ showed a clear decrease in cell clustering, which was not significantly increased in comparison to cell clustering mediated by APP. The above studies provide clear evidence that reduced APP shedding promotes trans-directed APP interaction.

Notably, inhibition of APP shedding was more pronounced in mammalian than in S2 cells, suggesting similar, but non-identical cleavage preferences. In *Drosophila*, an ADAM-like protease is encoded by the *Kuzbanian* gene (*KUZ*, Rooke et al., 1996), which was shown to cleave Notch (Pan and Rubin, 1997), APP and APPL at the expected α-secretase cleavage sites (Carmine-Simmen et al., 2009). In case of β-secretase the similarity between *Drosophila* and human cells seems to be lower. In mammals two closely related aspartic proteases, BACE1 (β-site APP-cleaving enzyme; Vassar et al., 1999) and BACE2 (Solans et al., 2000), have been identified. In *Drosophila*, two aspartic proteases were identified by their homology to human BACE (DASP1 and DASP2), but their β-secretase activity towards human APP or APPL discussed is controversial (Kotani et al., 2005; Carmine-Simmen et al., 2009; Poeck et al., 2012). For DASP2a it was reported that it cleaves APPL and APP in a BACE like manner at a position N-terminal of Asp1 (Aβ numbering) at the β-cleavage site, resulting in β-CTF- and Aβ peptide-like cleavage products with an higher apparent molecular weight (Carmine-Simmen et al., 2009; Poeck et al., 2012). Possibly, cleavage of APP by DASP2, the postulated *Drosophila* BACE homolog, is not affected by the APP mutants used in our analysis. This might explain the higher residual generation of sAPP in *Drosophila* compared to mammalian cells.

To investigate the consequences of APP processing we used a well-established mixed co-culture system assaying the synaptogenic activity of cell adhesion molecules (Scheiffele et al., 2000; Graf et al., 2004; Biederer and Scheiffele, 2007). Comparable to NLG-1, expression of APP in HEK293 cells potently promoted synaptic puncta formation (Figure 3) as reported before by Wang et al. (2009). Both investigated processing deficient APP forms showed a significantly higher amount of Synaptophysin puncta and also an elevated fractional cell area covered by Synaptophysin on the transfected HEK293 cells (Figure 3). These data clearly show that reduced APP processing enhances its synaptogenic activity to an even higher extent than NLG-1, a *bona fide* SAM (Scheiffele et al., 2000; Sudhof, 2008).

**Figure 4:**
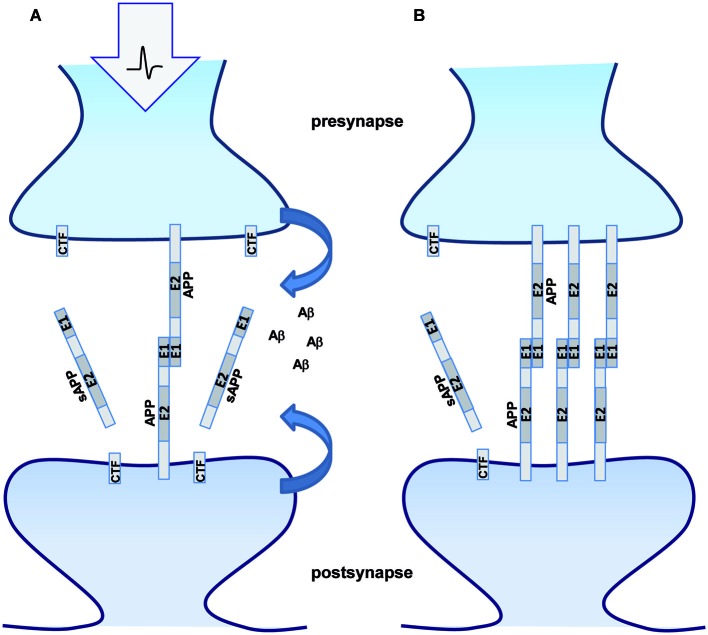
**APP processing modulates synaptic function**. Schematic representation of APP trans-dimerization and its synaptic function. **(A)** Enhanced synaptic activity leads to increased APP processing and less intact full length APP available for trans-dimerization at the synapse. The secreted cleavage product sAPPα and Aβ might bind different receptors at the pre- and postsynaptic sites modulating neurotransmission and/or synapse formation. **(B)** Under conditions of low synaptic activity less APP processing occurs and more full length APP will be available for trans-synaptic dimerization.

Similarly, it has recently been shown that NLG-1 undergoes ectodomain shedding by ADAM10 (Suzuki et al., 2012). Interestingly, neuronal activity increased shedding of NLG-1 and thus might negatively regulate remodeling of spines (Suzuki et al., 2012). In line with this, also Calsyntenin 3 is subject to ectodomain shedding (Araki et al., 2004), whereas membrane anchored Calsyntenin 3 displays a higher synaptogenic activity than its secreted form (Pettem et al., 2013). Comparable mechanism could be in place for APP, as overexpression of APP induces dendritic spine formation (Lee et al., 2010), whereas loss of APP goes along with a reduced spine density and LTP defects, which can be only partially rescued by sAPPα (Weyer et al., 2011). Interestingly, APP transgenic mice overexpressing human APP mutated at the α-secretase cleavage site show a phenotype of decreased sensitivity to NMDA, a sign of NMDA receptor hypofunction and misregulated synapse formation as well as epileptic seizures (Moechars et al., 1996, 1998). Furthermore, processing of APP and sAPP secretion was shown to be increased by synaptic activity (Farber et al., 1995). In turn, APP cleavage products also affect neurotransmission (Furukawa and Mattson, 1998; Kamenetz et al., 2003; Taylor et al., 2008; Weyer et al., 2011; Wilhelm et al., 2014). Although the complex interplay of these different functions of APP and its cleavage products is not yet understood, our data suggest that APP processing negatively regulates APP trans-dimerization and thus its cell adhesion properties and synaptogenic activity. Therefore it is tempting to speculate that the function of trans-interacting APP might dominate under conditions of low neuronal activity or in early steps of synaptogenesis, whereas activity-dependent increase of APP processing might cause a domination of APP cleavage product function (Figure 4). Formation of the chemical synapse is a multi-step process involving target recognition followed by inductive interactions that result in recruitment of a special set of synaptic proteins, such as receptors, signaling molecules and organelles. A large set of diverse synaptic cell adhesion molecules has been implicated in this process, including proto-cadherins, cadherins, neural cell adhesion molecule (NCAM), L1 family CAMs, synaptic immunoglobulin superfamily members, NLG-1/Neurexin complexes, Integrin/extra cellular matrix complexes (Dalva et al., 2007; Missler et al., 2012). How this complex network is orchestrated and precisely how APP and its homologs as well as the different cleavage products fit into the SAM machinery will therefore be of high interest for future studies.

## Materials and methods

### Cloning of APP-F615P and APPΔF616 and -ΔS622, APP-D8

pBluescriptSK(+) APP695 NT-myc (Soba et al., 2005) was used as a template and the constructs were generated by PCR using appropriate mutagenesis primer containing a BglII restriction site. For cloning of the different APP constructs the resulting PCR products were ligated into a predigested pSK(+)-APP695 NT-myc vector. The constructs were further subcloned into pUAST and pCEP4 vectors. The identity of all constructs was confirmed by double stranded sequencing.

E1 domain deletion constructs were cloned from the corresponding pUAST-APP-FL clones (pUAST-APP-D8 and pUAST-APP-F615P) with the pUAST-APPΔE1 clone (Soba et al., 2005) and pUAST in a three-fragment-ligation. The NotI/EcoRI-fragment from pUAST-APPΔE1 together with the XhoI/EcoRI-fragment of pUAST-APP-D8/-F615P was ligated into NotI/XhoI digested pUAST-vector. The corresponding pUAST clones were digested NotI/EcoRI and EcoRI/XhoI. The two fragments of interest were ligated into NotI/XhoI digested pcDNA3.1-Zeo vector in a three-fragment-ligation.

APPΔ622 contains a deletion of Aβ_minus5_–Aβ_24_ of the Aβ domain (APPΔ592–621) (APP695 numbering) including the β-cleavage site at position 1 and the α-cleavage site at position 17 within the Aβ sequence. APPΔF615 contains a deletion of sequence Aβ_minus5_–Aβ_19_ within the Aβ domain, which comprises the α- as well as the β-cleavage site of APP (APPΔ592–621). APP-D8 contains a deletion of sequence Aβ_10_–Aβ_24_ within the Aβ domain, which was replaced by eight aspartates, comprising only the α-cleavage site. In APPΔE1, the N-terminal sequence aa 31–192 is deleted. The identity of all constructs was confirmed by double stranded sequencing. The expression vector pMT-Kuz^DN^, encoding a dominant negative form of Kuzbanian was provided by the *Drosophila* genomic resource center.

### Antibodies

Monoclonal antibodies used in this study: anti-APP 22C11 (epitope hAPP aa 66–81) (Weidemann et al., 1989) for detection of sAPP_total_. Antibody W02 (Ida et al., 1996) was used for detection of sAPPα. Mouse monoclonal β-actin antibody was from Sigma. Rabbit Monoclonal antibody against the APP C-terminus (Y188) was from Epitomics. The polyclonal sAPPα antibody recognizes the ISEVKM sequence at the C-terminus of wild type human sAPP (Immuno-Biological Laboratories, Inc., Minneapolis, MN, USA) (Eggert et al., 2009). Anti-c-myc rabbit polyclonal antibody was from Santa Cruz for detection of cell surface biotinylated proteins. Rabbit polyclonal antibody 22734 with an epitope in the APP ectodomain (Prof. Dr. Multhaup), anti-c-myc, rat (JAC6) (Serotec) was used for detection of myc-tagged proteins via immunocytochemistry. MAP2 rabbit polyclonal antibody was from Santa Cruz, TAU1 mouse monoclonal antibody from Chemicon, Synaptophysin mouse monoclonal antibody was from Sigma, Synaptophysin guinea pig polyclonal from Synaptic systems, SV2 mouse monoclonal antibody from Developmental Studies Hybridoma bank.

For Enhanced Chemical Luminescence (ECL) detection after Western blotting (Pierce), appropriate Horseradish-Peroxidase (HRP) coupled anti-mouse, anti-rabbit, or anti-rat antibodies (Jackson Immunoresearch) were used. Alexa-488, Alexa-594 and Alexa-647 (Molecular Probes) were used as secondary antibodies.

### Cultivation of semi-adherent Schneider (S2) cells

S2 cells (Schneider, 1972) were cultivated in T-75 cell culture flasks (Costar) in 20 ml growth medium (Schneider’s Medium (Invitrogen), 10% FBS, 1% penicillin/streptomycin). S2 cells stayed adherent until 70–80% confluency was reached, after which they detached and proliferated in suspension. At this time point, cells were passaged by resuspending until a single cell suspension was present. Cells were cultivated at 25°C under a normal atmosphere.

### Transient transfection of Schneider (S2) cells

S2 cells were plated in 12 well dishes at 30–40% confluency the day before transfection in 2.5 ml total volume. 2–3 h before transfection, the growth medium was replaced with 800 μl of fresh medium, and cells were transfected with Effectene (Qiagen) according to the manufacturer’s protocol. In case of co-transfecting several plasmids, equal DNA amounts were used. Expression was induced by adding CuSO_4_ (0.5 M stock in ddH_2_O) to a final concentration of 500 μM.

### Analysis of sAPP_total_ secretion in Schneider cells

Sixteen hour after induction of heterologous protein expression in S2 cells, medium was replaced by 1 ml fresh medium and conditioned for 3 h. Afterwards, the medium was collected and cleared from cell debris by centrifugation at 10.000 × g for 10 min. The cells were directly lyzed in 100 μl 2 × SDS sample-buffer supplemented with Benzonase (Merck) and denatured at 95°C for 5 min. sAPP_total_ was immunoprecipitated from the supernatant with an anti-APP antibody (22734, epitope APP ectodomain). Concentrated supernatant and cell-lysate samples were analyzed by SDS-PAGE and immunoblotted with antibody 22C11. Films were digitized and signals were quantified densitometrically using Image Gauge (Fuji Systems). Relative ratios of sAPP_total_ compared to cellular APP were calculated for all tested constructs.

### Schneider cell aggregation assay

Sixteen hour after induction of heterologous protein expression, S2 cells were centrifuged at 800× g for 5 min. The supernatant was replaced by 1 ml fresh growth medium (containing 10% FCS, 1% pen/strep) and cells were resuspended to obtain single-cell-suspensions. Cells were counted using a Neubauer Counting Chamber. For analysis, 4 × 10^5^ cells in single cell suspension were aggregated in a total volume of 1 ml fresh growth medium in a 24-well plate for 2 h at 90 rpm on a horizontal shaker (Greiner 24-well suspension culture dish). Afterwards, cells were transferred to poly-L-lysine coated cover slips and allowed to attach for 2–3 h. Aggregated Schneider cells were further processed by immunocytochemistry.

### Immunocytochemistry and quantification of aggregated S2 cells

Aggregated S2 cells attached to cover slips were fixed in 4% PFA for 10 min. Cells were washed three times with PBS and permeabilized with 0.1% NP40/PBS for 10 min. Afterwards, cells were washed again three times in PBS, blocked with 5% normal goat serum in PBS for 1 h and stained over night at 4°C with a rat anti-c-myc-antibody (JAC6, Serotec). Anti-rat AlexaFluor488 (Invitrogen) was used as secondary antibody for 1 h at room temperature. After extensive washing, cells were embedded in Mowiol.

For quantification of aggregated cells, clusters of three or more transfected cells were scored as positive. In total, 500–1100 transfected cells from at least five independent experiments were counted for each experimental setup in a blinded fashion. Clusters of non-transfected cells were not counted. The average transfection efficiency varied between 10% and 30%. To minimize variation, only experiments with similar transfection rates (+/− 10%) were compared with each other. Statistical significance of all quantified experiments was tested with a Students *T*-test, *p* < 0.05 was designated as significant, *p* < 0.001 as highly significant.

### Co-culture assay

Co-cultures of HEK293 cells and neurons were performed essentially as described (Biederer and Scheiffele, 2007). HEK293 cells were cultured in DMEM-High glucose supplemented with 10% fetal bovine serum and 1% Pen/Strep. Cortical neuron cultures were prepared from E14 mouse embryos (C57/Bl6J) according to previously described protocols (Biederer and Scheiffele, 2007). Briefly, cortices were dissected in ice cold HBSS supplemented with 10 mM HEPES. After a 15 min incubation time at 37°C in 0.05% Trypsin-EDTA, the cortices were washed five times in 1× HBSS and triturated with a pasteur pipette. The dissociated cells were resuspended in DB1 media [DMEM with 10% (v/v) FCS, 0.79% (w/v) D-glucose, and 2 mM glutamine] and plated onto 14 mm coverslips pretreated with poly-L-lysine (20 μg/ml in borate buffer). After 6 h, the media was replaced with NM media (Neurobasal medium with 2% (v/v) B-27 supplement, and 2 mM Glutamax). At DIV 6 of the neuronal culture, HEK293 cells were transfected with jetPRIME (Polyplus) according to manufacturer’s instructions. *GFP* pcDNA3.1+ was used as a negative control, NLG-1-1 HA pcDNA3.1+ as a positive control. Constructs NT my APP695 wt, NT my APP695 D8 pcDNA3.1+ and NT my APP695 Δ622 pcDNA3.1+ were analyzed in addition. Twenty-four hours later, transfected cells were seeded onto the cultured primary neurons (DIV7). After 24 h (DIV8), the cultures were fixed in 4% (w/v) PFA/PBS supplemented with 4% (w/v) sucrose, permeabilized in 0.1% NP401× PBS. The potentially synapse inducing proteins expressed in HEK293 cells were detected with anti-c-my (1:200, Serotec) and anti-HA (1:300, Roche) antibodies (secondary antibody Alexa-Flour 488, Invitrogen). In addition the cultures were stained with the presynaptic marker Synaptophysin (1:200, Sigma) to visualize the synaptic puncta (secondary antibody Alexa-Flour 594, Invitrogen). Changes in the number of synaptic puncta or the area covered by synaptic puncta were used as a readout of induced presynaptic differentiation. Furthermore, antibody MAP2 (Santa Cruz) was used as a dendritic marker to ensure correct quantitative analysis of HEK293/axonal contacts. Only Synaptophysin puncta at MAP2 negative HEK cells were analyzed at so called hemisynapses between HEK cells and axons, not dendrites and axons. Z-stack images were taken with the microscope Axio Observer Z.1 (Zeiss) (apotome) and quantification was performed via ImageJ analysis according to an already established protocol (Fogel et al., 2007). Statistical analysis was performed with one-way factorial ANOVA followed by Tukey’s *post hoc* analyses (*n* ≥ 4; * *p* < 0.05; ** *p* < 0.01; *** *p* < 0.001). Results are presented as mean ± SEM.

### Cell surface biotinylation

HEK293 cells were seeded in a 6 well plate at a density of 3 × 10^5^ cells per well and transfected the following day with Lipofectamine 2000 (Invitrogen) according to manufacturer’s instructions. To examine surface levels of APP, cells were rinsed two times with ice cold 1× PBS. Cell surface proteins were biotinylated with 1 ml EZ-Link Sulfo-NHS-LC-Biotin (Pierce) (2 mg/ml) in ice cold PBS for 30 min at 4°C. Cells were washed three times with ice-cold 1 × PBS containing 100 mM glycine to quench unconjugated biotin and lyzed in 1 × RIPA buffer (20 mM Tris/HCl pH 8.0, 150 mM NaCl, 1% NP-40 (w/v), 0.5% deoxycholate, 5 mM EDTA pH 8.0, 0.1% SDS.) including protease inhibitors (Roche). 20 μg protein of cell lysate was used for the direct load. Equal amounts of protein were incubated with NeutrAvidin Agarose Resin (Pierce) over night at 4°C. Biotinylated proteins were recovered by boiling in 2× sample buffer with DTT for 5 min at 95°C and separated on 8% Tris/glycine gels and detected with antibody c-myc.

### Analysis of APP processing in HEK293 cells

HEK293 cells were cultured in DMEM-high glucose supplemented with 10% FBS and 1% penicillin/streptomycin and seeded in a 6 well plate at a density of 3 × 10^5^ cells per well and transfected the following day with Lipofectamine 2000 (Invitrogen) according to manufacturer’s instructions. Medium was changed after 4 h and conditioned for 17 h overnight. For analysis the media was centrifuged for 10 min at 13.000× g at 4°C. The supernatant was used for analysis of sAPP_total_, sAPPα, sAPPβ by Western Blot with the respective antibodies.

The cells were harvested in ice cold 1× PBS and subsequently incubated for 15 min in lysis buffer (50 mM Tris/HCl, pH 7.5, 150 mM NaCl, 5 mM EDTA, and 1% Nonidet P40) supplemented with protease inhibitors (Roche) at 4°C. After a 10 min centrifugation step at 12.000× g at 4°C, the supernatants were collected and the protein concentration was determined with a BCA assay (Sigma). Equal amounts of protein were separated by PAGE and subjected to Western Blot analysis of full length APP. Densitometric quantification of the Western Blots was performed with Image J software. Statistical analysis was performed with one-way factorial ANOVA followed by Tukey’s *post hoc* analyses (*n* ≥ 5; * *p* < 0.05; ** *p* < 0.01; *** *p* < 0.001). Results are presented as mean ± SEM.

## Author contributions

Ronny Stahl, Sandra Schilling, Peter Soba, Carsten Rupp, Katja Wagner performed different experiments and analyzed the data. Tobias Hartmann, Gunter Merdes provided material and analyzed data. Simone Eggert and Stefan Kins designed experiments, wrote the manuscript.

## Conflict of interest statement

The authors declare that the research was conducted in the absence of any commercial or financial relationships that could be construed as a potential conflict of interest.
